# Hospital Employees’ Well-Being Six Months after the COVID-19 Outbreak: Results from a Psychological Screening Program in Italy

**DOI:** 10.3390/ijerph18115649

**Published:** 2021-05-25

**Authors:** Giulia Lamiani, Lidia Borghi, Silvia Poli, Katia Razzini, Claudio Colosio, Elena Vegni

**Affiliations:** 1 Department of Health Sciences, University of Milan, 20142 Milan, Italy; lidia.borghi@unimi.it (L.B.); claudio.colosio@unimi.it (C.C.); elena.vegni@unimi.it (E.V.); 2 Department of Neurosciences, Biomedicine and Movement Sciences, University of Verona, 37134 Verona, Italy; silvia.poli@univr.it; 3 Prevention and Protection Service, ASST Santi Paolo e Carlo, 20142 Milan, Italy; katia.razzini@asst-santipaolocarlo.it; 4 Occupational Health Unit, ASST Santi Paolo e Carlo, 20142 Milan, Italy; 5 Clinical Psychology Unit, ASST Santi Paolo e Carlo, 20142 Milan, Italy

**Keywords:** coronavirus, mental health, psychological distress, hospital workers, healthcare workers, PTSD, anxiety, depression, preventive interventions, clinical psychology

## Abstract

The COVID-19 outbreak has taken a heavy toll on the mental well-being of healthcare workers. This study aims to describe a psychological screening program developed at a large University Hospital in Milan, Italy, and assess the psychological outcomes of employees and associated factors. A survey was electronically conducted among hospital employees between July and October 2020. Sociodemographic data, information about COVID-19 experience and three scales assessing anxiety (STAI-Y1), depression (HAM-D) and post-traumatic stress disorder (PCL-5) were collected. A total of 308 employees (80% women; mean age 45.1 years) responded: 16% physicians, 68% other healthcare professionals, and 16% administrative staff. Employees reported moderate/severe symptoms of anxiety (23%), depression (53%), and post-traumatic stress disorder (40%). At multivariate logistic regression analysis, having suffered a loss for COVID-19 in the personal context was independently associated with higher risk of moderate/severe anxiety (OR = 2.40; 95% CI 1.16–4.98), being female was associated with higher risk of moderate/severe depression (OR = 2.82; 95% CI 1.43–5.59), and having had a family member affected by COVID-19 was associated with higher risk of moderate/severe post-traumatic stress disorder (OR = 2.75; 95% CI 1.01–7.48). COVID-19 personal experience may have a profound impact on hospital workers’ mental health and should be considered in supportive interventions.

## 1. Introduction

The novel coronavirus causing the Coronavirus disease 2019 (COVID-19) was declared a global pandemic by the World Health Organization on 11 March 2020. Since then, the management of the COVID-19 outbreak has proved to be a great challenge for healthcare systems and healthcare workers worldwide [1]. All the literature produced during these last months of the pandemic has almost unanimously showed that the COVID-19 pandemic has put healthcare workers at a higher risk of developing negative psychological outcomes such as anxiety, depression, insomnia, and post-traumatic reactions [2,3,4].

Some studies explored the psychological impact on healthcare workers based on their degree of exposure to the COVID-19 virus. These studies highlighted that healthcare workers directly involved in the care of COVID-19 patients have presented a greater risk of developing mental health symptoms compared to those who were not [5,6,7,8,9,10]. The long and burdensome shifts, the need to provide emotional support to patients in complete isolation, the frequent deaths, and the demands in taking difficult clinical and ethical decisions might have contributed to increase psychological distress in frontline healthcare workers. Nevertheless, other studies highlighted that even non-frontline healthcare workers showed high levels of psychological distress [11,12,13], as they might have felt sidelined with a sense of helplessness in the face of the COVID-19 crisis, and not able to care properly for non-COVID-19 patients [14] due to the delay in treatments and a decrease of non-COVID-19 patients’ hospital admissions.

Other studies that compared different hospital workers’ categories reported contradictory or inconsistent results. Some research has found that medical staff (including doctors and nurses) report greater psychological distress than administrative staff [6] probably because of the intense and close exposure to COVID-19 patients. Consistently, two recent reviews [15,16] reported higher psychological distress among frontline workers and nurses. However, other studies [17] that compared medical workers (including physicians and nurses) to non-medical workers (including allied health professionals, pharmacists, technicians, administrators, clerical staff, and maintenance workers), showed that non-medical healthcare workers reported a higher prevalence of anxiety. Consistently with these findings, Li and colleagues (2020) [18] hypothesized that vicarious traumatization could explain the psychological distress of non-frontline nurses and the general public which they found in their study.

In addition to the profession and the level of exposure to COVID-19 patients, preliminary studies suggests that personal COVID-19 experiences, such as self-quarantine, fear of being infected, fear of transmitting the disease to family members and subsequent feelings of guilt and remorse may also have constituted risk factors for healthcare workers’ mental health [19,20]. The diffusivity and severity of COVID-19 pandemic threatened healthcare workers’ sense of being magically immune from their patients’ illnesses and exposed them to the same fears of the general population. Many healthcare workers were infected by COVID-19, some lived in fear of infecting their family members, and others experienced the loss of colleagues or family members. Healthcare workers had limited opportunities to distance themselves from the pandemic as it pervaded everyday aspects of their professional and personal life [21]. However, the influence of healthcare workers’ personal COVID-19 experiences (such as being affected by COVID-19 or having had a family member affected by COVID-19) in determining their mental health has not been explored in the literature so far.

Given the diffusivity and severity of COVID-19 infection, the literature is unanimous in suggesting that the psychological well-being of hospital workers during the pandemic should deserve a specific clinical attention. Healthcare workers are generally reluctant in seeking psychological support and might find it hard to recognize their own psychological distress [22,23]. For this reason, it is essential for healthcare organizations engaged in the fight against COVID-19 to plan proactive interventions in supporting the psychological well-being of their employees [24,25,26].

In order to address this need, a psychological screening program named “Well-being Project” was implemented in a large University Hospital in Milan, Italy. This project was designed to monitor and sustain the psychological well-being of the hospital employees at the beginning of summer 2020, which coincided, in Italy, with the end of the first pandemic wave. This study aims to: (1) describe the psychological screening program named “Well-being Project”; and (2) assess the prevalence of anxiety, depression, and post-traumatic stress disorder among hospital employees and their contributing factors. Among contributing factors, we collected data on professional and personal COVID-19 experiences because of their importance in affecting employees’ well-being.

## 2. Materials and Methods

### 2.1. The Well-Being Project

In order to monitor employees’ psychological well-being after the first wave of the COVID-19 pandemic, the Unit of Clinical Psychology of the Santi Paolo and Carlo Hospital, Milan, Italy, implemented the “Well-being Project” in collaboration with the Prevention and Protection Service and the Occupational Health Unit. This project aimed to monitor employees’ well-being and provide them with supportive resources. The project consisted in a survey which was made available to all hospital employees through the hospital e-learning platform. The survey included a sociodemographic section where participants were asked about their sex, age, profession, if they had contact with COVID-19 patients, if they had COVID-19, if they had a family member affected by COVID-19, if they experienced losses for COVID-19 in the personal context and if they experienced losses for COVID-19 at work. In addition, the survey included three questionnaires assessing anxiety, depression, and post-traumatic stress disorder, respectively. At the end of each questionnaire, a visual alert (green, yellow, or red-alert) appeared on the screen along with a feedback message on the level of personal psychological well-being. The contact information of the Clinical Psychology Unit appeared on the screen in case the employees wanted to discuss the survey results. Upon completion of all the questionnaires, four audio-recorded mindfulness practices for stress reduction were offered to all participants for download (Table 1).

### 2.2. Participants

The invitation to the survey was sent by email to all Santi Paolo and Carlo Hospital employees (*n* = 3974). Santi Paolo and Carlo Hospital is a public hospital of the Lombardy Region, serving the south and west area of Milan. The survey opened on 14 July and closed on 13 October 2020. During this timeframe, two reminders were sent by email to all employees.

### 2.3. Measures

#### 2.3.1. Anxiety

Anxiety was assessed by the State-Trait Anxiety Inventory-Y (STAI-Y) using the State Anxiety form (STAI-Y1) [27]. The STAI-Y1 measures a participant’s state of anxiety, which is the transitory state of fear and emotional tension in response to a perceived threatening situation. The STAI-Y1 includes 20 items on a 4-points Likert scale ranging from 1 (Not at all) to 4 (Very much); 10 items have a reverse score. Items are summed to provide a total state anxiety score, which ranges from 20 to 80. Higher scores indicate higher levels of state anxiety. In the survey, we used the Italian validated version of STAI-Y1 [28]. As no cut off scores have been validated, we categorized the total score starting from the mean and standard deviation (SD) of the Italian population [28]. For females, we considered green a total score within one standard deviation (SD = 11) from the mean (39.93), yellow a score between one and two SDs from the mean (50.94–61.93), and red a score over two SDs from the mean (≥61.94). For males, we considered green a total score within one SD (9.7) from the mean (36), yellow a score between one and two SDs from the mean (45.8–55.4) and red a score over two SDs from the mean (≥55.5) [29]. The value of clinical threshold was set at the lower limit of the yellow interval. Cronbach Alpha of STAI-Y1 in this study was 0.95.

#### 2.3.2. Depression

Hamilton Depression Scale (HAM-D) was used to assess depression [30]. HAM-D is a self-reported scale used to measure the severity of depressive symptoms. It is not used to diagnose the presence or absence of a depressive disorder. In this study, we used the Italian version of the original HAM-D, which is composed of 17 items [31]. Seven items are graduated on 3-point Likert scales ranging from 0–2; 9 items are graduated on 5-point Likert scales ranging from 0 to 4 and 1 item is graduated on a 4-point Likert scale ranging from 0 to 3. Each point of the Likert scale is associated with a precise description of symptoms assessed. Items are summed to provide a total score, which ranges from 0 to 53. Higher scores indicate greater pervasiveness of depressive symptoms. Based on the literature [32], we considered a total score between 0 and 7 as indicative of absence of depressive symptoms (green), a score between 8 and 17 as indicative of mild depressive symptoms (yellow) and a score ≥18 as indicative of moderate or severe depressive symptoms (red). The value of the clinical threshold was set at the lower limit of the yellow interval. Cronbach Alpha of HAM-D in this study was 0.79.

#### 2.3.3. Post-Traumatic Stress Disorder

PTSD Checklist for DSM-5 (PCL-5) was used to assess Post-Traumatic Stress Disorder (PTSD). PCL-5 is widely used to detect significant symptoms of PTSD but not to make a clinical diagnosis of PTSD. PCL-5 has been validated and presented good psychometric properties [33]. PCL-5 is composed of 20 items assessing PTSD symptoms in accordance with the DSM-5 criterions [34]: exposure to actual or threatened death, serious injury, or sexual violence (criterion A); intrusion symptoms (criterion B); avoidance of stimuli (criterion C); negative alterations in cognitions and mood (criterion D); and marked alterations in arousal and reactivity associated with the traumatic event (criterion E). In this study, we used the PCL-5 Standard form without criterion A component [35]. Respondents are asked to rate how bothered they have been by each of the 20 items in the past month on a 5-point Likert scale ranging from 0 (Not at all) to 4 (Extremely). Items are summed to provide a total severity score, which ranges from 0 to 80. Higher scores indicate higher levels of PTSD. Literature agrees to consider a total score ≥31 as indicative of probable PTSD [36]. However, a provisional PTSD diagnosis can be made by counting all symptoms rated as 2 or more as present, and then following the DSM-5 diagnostic rule which requires the presence of at least 1 B item (questions 1–5), 1 C item (questions 6–7), 2 D items (questions 8–14), 2 E items (questions 15–20) rated ≥2 for PTSD to be diagnosed (US Department of Veterans Affairs, 2020). Based on this information, we considered a total score between 0 and 11 as indicative of absence of PTSD (green), a score between 12 and 30 as indicative of possible PTSD (yellow) and a score ≥31 as indicative of probable PTSD (red). The value of clinical threshold was set at the lower limit of the yellow interval. The Cronbach Alpha of PCL-5 in this study was 0.95.

### 2.4. Statistical Analysis

Descriptive data were reported as mean (SD), median (range) or number of observations (percentage). The Kolmogorov-Smirnov test was used to assess the normality of the distribution of the continuous variables. Cronbach Alpha was calculated for each scale to assess internal consistency. Association among STAI-Y1, HAM-D, and PCL-5 was assessed using Pearson r or Spearman ρ when appropriate. STAI-Y1, HAM-D, and PCL-5 were then dichotomized into 0 and 1, where 0 indicated a total score under the clinical threshold and 1 indicated a score equal or over the clinical threshold. To assess association between psychological outcomes and sociodemographic characteristics and COVID-19 experience, bivariate and multivariate logistic regression analyses were performed separately, considering dichotomized STAI-Y1, HAM-D, and PCL-5 as dependent variables. Sex, age, profession, having had contact with COVID-19 patients, having had COVID-19, having had a family member affected by COVID-19, having experienced losses for COVID-19 in the personal context and having experienced losses for COVID-19 at work were entered in the models as covariates. Profession was discretized into three categories: administrative staff, physicians, and other healthcare professionals (including nurses, allied healthcare professionals, psychologists, physiotherapists, health and safety officers, and biologists/lab technicians). The odds ratios (OR) and related 95% confidence interval (CI) were calculated. The significance level was set to *p* < 0.05 (two-tailed test). All the statistical analyses were carried out through the IBM SPSS Statistics for Windows, Version 26.0 [37].

### 2.5. Ethics

The study was conducted according to the guidelines of the Declaration of Helsinki and was approved by the Ethical Committee of Santi Paolo and Carlo Hospital Milan with the register number 2020/ST/422. All participants signed an electronic informed consent before the questionnaires administration. The consent was composed of two parts. In the first part, participants expressed their consent for the data to be used for research purposes in an anonymized and aggregated form. In the second part, participants expressed their consent to transmit their data to the Unit of Clinical Psychology and be contacted by a psychologist to discuss the results.

## 3. Results

### 3.1. Paticipants

A total of 308 employees participated in the project (8% response rate). The sociodemographic characteristics and COVID-19 experience of responding employees are reported in Table 2.

### 3.2. Anxiety, Depression, and Post-Traumatic Stress Disorder

**Figure 1:**
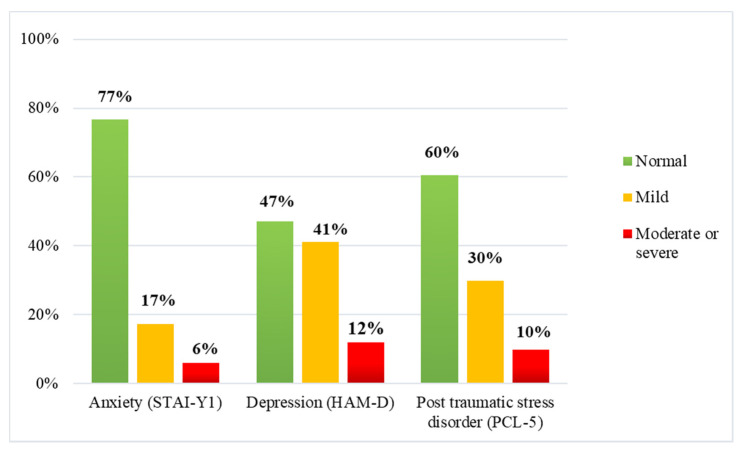
Percentage distribution of employees according to clinical threshold for the psychological outcomes.

Of the participating employees, 306 (99%) completed the STAI-Y1, 253 (82%) completed the HAM-D and 238 (77%) completed the PCL-5. As for STAI-Y1, the mean (SD) was 41.59 (11.01), and the median (range) was 39 (20–75). As for HAM-D, the mean (SD) was 9.20 (6.31), and the median (range) was 8 (2–32). As for PCL-5, the mean (SD) was 12.69 (12.97), and the median (range) was 9 (0–72). The total scores of STAI-Y1, HAM-D and PCL-5 were all positively associated. Correlation coefficient of STAY-Y1 with HAM-D was *r* = 0.62 (*p* < 0.001) and with PCL-5 was *ρ* = 0.73 (*p* < 0.001). Correlation coefficient of HAM-D with PCL-5 was *ρ* = 0.69 (*p* < 0.001). Figure 1 shows the percentages of employees who received a green, yellow, or red alert.

Specifically, on the STAI-Y1 scale 71 employees (23%) received a yellow or red alert, reporting mild (17%) or moderate/severe symptoms (6%). On the HAM-D scale, 134 employees (53%) received a yellow or red alert, reporting mild (41%) or moderate/severe (12%) depressive symptoms. On the PCL-5 scale, 94 employees (40%) received a yellow or red alert, reporting a possible (30%) or a probable PTSD (10%).

Table 3 reports results from univariate and multivariate logistic regression analyses. At univariate analysis, STAY-Y1 was associated with having experienced losses for COVID-19 in the personal context (*p* = 0.003). HAM-D was associated with being female (*p* = 0.002). PCL-5 was associated with having had contact with COVID-19 patients (*p* = 0.044), having had COVID-19 (*p* = 0.033), having had a family member affected by COVID-19 (*p* = 0.010), and having experienced losses for COVID-19 at work (*p* = 0.013).

In the multivariate models, STAY-Y1 remained associated with having experienced losses for COVID-19 in the personal context (*p* = 0.018). HAM-D remained associated with being female (*p* = 0.003) and PCL-5 remained associated only with having had a family member affected by COVID-19 (*p* = 0.048). Having experienced losses for COVID-19 at work did not reach the statistical significance (*p* = 0.064).

### 3.3. Clinical Support

All participating employees but one (*n* = 307) gave their consent to be contacted by the Unit of Clinical Psychology. Of these, 52 (17%) reported one or more than one red alerts and were contacted by phone by a psychologist of the Unit of Clinical Psychology for a preliminary assessment. Of the employees contacted, 36 (70%) agreed upon and received a psychological consultation, 8 (15%) stated they were already receiving psychological support and 8 (15%) declined an appointment.

## 4. Discussion

This study is one of the first attempts to describe a program of screening and intervention designed to promote hospital employees’ psychological well-being after the first wave of COVID-19 pandemic. Several psychological interventions have been developed during the emergency phase of the pandemic in order to decrease stress and prevent major psychological problems among hospital workers [38,39]. However, little is known about the kind of psychological interventions that have been put in place towards the monitoring of hospital employees’ psychological well-being right after the emergency phase. We know that during the emergency phase, the mental and physical resources, generally, tend to be allotted towards “doing”. Only once the emergency phase subsides does it become possible for hospital workers to reflect upon what happened and get in touch with their emotional experiences [40]. For this reason, we decided to implement a psychological screening program immediately after the emergency phase, hoping to enhance hospital employees’ self-awareness and provide them with resources to take care of their mental health.

The screening program focused on anxiety, depression and post-traumatic stress disorder, which have been the most commonly investigated outcomes in the existing literature. Our sample reported a notable prevalence of moderate/severe depression (53%) and PTSD (40%), and a lower frequency of moderate/severe state anxiety (23%). Our results are only partially consistent with the existing literature on psychological outcomes of healthcare workers conducted during or immediately after the emergency phase of the pandemic. A review [15] conducted during the first pandemic wave (data search on 6th May) highlighted a great variability of the prevalence of depressive and anxiety symptoms among healthcare workers, which ranged between 9% to 50% for anxiety and between 15% to 45% for depression. Another study [41] conducted in three large hospitals in Northern Italy during the first pandemic wave reported that 71% of healthcare professionals showed clinical levels of state anxiety, 37% reported clinical levels of post-traumatic stress, and 27% showed clinical levels of depression. Another Italian study [10], conducted during the emergency phase, found a lower prevalence of anxiety (18–21%) and a similar prevalence of depression (28–20%) among front-line and second-line healthcare workers.

Our study was conducted when the emergency phase of the first wave of the pandemic (that in Italy was between 8 March and 3 May 2020) was over and the country was lifting restrictions and reopening its social and commercial activities. This contextual factor might explain the lower prevalence of anxiety compared to depression and PTSD. At the same time, the higher percentage of employees reporting moderate/severe depressive and PTSD symptoms may suggest a persistence of psychological distress after the emergency phase and a clinical shift in the psychological symptoms reported. Our results are consistent with the literature on Disaster Psychology which describes the disillusionment phase as the moment where, after the heroic and honeymoon phases, it is possible to come to terms with the reality of the situation and start working through grief [42]. Indeed individuals may have lost not only loved ones and colleagues, but also dreams, freedom, and assumptions about life. Similarly, our findings are consistent with psychiatric literature that positions the onset of PTSD symptoms within three months of the trauma, but states that symptoms may also appear later than that and often persist for months after the initial trauma [34].

The literature suggests that grief reactions and post-traumatic stress are natural and adaptive responses and a normal part of the recovery process from a disaster [43,44]. However, our study identified some personal risk factors that are independently associated with a psychological response over the clinical thresholds in hospital employees. Anxiety of clinical relevance was associated with having experienced losses for COVID-19 in the personal context, independently from the other variables examined. Similar feelings of hyper-arousal and anguish were found among family members who lost a loved one for COVID-19 in the hospital [45]. Such anxiety may be due to the factors that characterize the deaths’ experience for COVID-19, such as the abrupt separation from the patient, the isolation, and the impossibility to see the patient’s body and to hold funeral rituals. These factors may hinder the representation and elaboration of the loss, thus leading to anxiety symptoms that may be suggestive of complicated grief [46].

Depressive symptoms of clinical relevance were associated only with female gender, independently from the other variables examined. Female gender seems to be a risk factor for depression, but not for anxiety and PTSD. Inconsistencies regarding the role of female gender as an independent risk factors for psychological distress in healthcare workers during the COVID-19 pandemic have been found [2,15]. However, the scientific literature on gender differences in psychopathology has shown a greater incidence of depression and anxiety in women compared to men [47,48], hence confirming an association of female gender with depression.

PTSD symptoms of clinical relevance were associated only with having had a family member affected by COVID-19. Interestingly, having experienced losses for COVID-19 at work emerged as an important factor related to PTSD, but did not reach the statistical significance. The exposure to numerous and frequent deaths of patients and the consequent sense of helplessness may have had a role in the development of post-traumatic symptoms. However, in our study this aspect did not contribute to PTSD as much as the personal experience of having had a family member affected by COVID-19. The experience of having a family member affected by COVID-19 may have contributed to blurring the boundaries between the personal and professional areas, thus facilitating the identification of hospital workers with their patients/families. In this sense, working in the hospital and caring for COVID-19 patients could be a trigger of personal unpleasant experiences related to COVID-19. Another hypothesis is that the idea of possibly having had a role in the infection of a family member, often reported in the literature [49], might have increased the feelings of blame and guilt and negative beliefs about oneself, all factors that are part of the PTSD experience. Mental health professionals should address the possible COVID-19 related guilt when working with hospital workers [50].

Our study presents some limitations. Despite the screening was anonymous, the survey received a low response rate. This might limit the generalizability of the results, as there could be a selection bias. Interestingly, nurses were the most represented in the respondents’ sample. Administrative staff, physicians and psychologists were almost equally represented in the survey despite their different proportion in the hospital workforce and their different involvement in the care of COVID-19 patients. This may suggest that participants who completed the survey might have been those presenting more distress or those more sensible to psychological issues.

Several factors may have contributed to the low response rate. Due to budget constraints, participants did not receive any incentives to complete the survey. The survey was conducted during the summer, a time where normally employees are less attentive to work-related activities, and was sent by email just like many other institutional communications. In addition, hospital employees may have been reluctant to engage in a survey investigating their psychological well-being. It is known that healthcare professionals are often self-reliant and tend not to ask for help [23,51]. The hero narrative that was promoted in the media during the first wave of COVID-19 pandemic could have reinforced this tendency. Emphasizing idealization and self-sacrifice, the hero narrative might have increased the stigma on help-seeking behaviors [22,52], thus reducing the motivation to engage in a psychological screening program. Another limitation concerns the death rate of the sample across the three questionnaires, which should be taken into account when interpreting the results. Finally, the cross-sectional design of the study limits the inference on the causality of the relationship between sociodemographic characteristics, COVID-19 experience, and the psychological outcomes. Longitudinal studies should be implemented to assess the psychological outcomes at different time points of the COVID-19 pandemic.

## 5. Conclusions

This study is one of the first to describe a psychological screening program for hospital workers six months after the COVID-19 outbreak. The program testifies the attention of the organization to the mental well-being of its workers and offers an example of a proactive psychological intervention that could be implemented to reach out to hospital workers who may present a psychological suffering. This study has also the merit of having assessed COVID-19 related personal risk factors and not only professional risk factors. The study identified specific personal risk factors that should be monitored in the near future, such as the presence of personal losses for COVID-19, the presence of family members affected by COVID-19 and female gender. Psychological support should address issues related to bereavement and post-traumatic stress, which may arise after the pandemic emergency phase. Organizational interventions should be promoted to decrease the stigma around help-seeking behaviors among healthcare professionals and sustain their motivation.

## Figures and Tables

**Table 1:** Mindful practices for hospital employees.

Title of Mindful Practice	Description Offered to Employees	Duration of Practice (Minutes)
Practice of letting go.	This practice is useful to lighten your mind. The practice of letting go is useful for overcoming the crowding of involuntary thoughts and feelings that weigh on the mind, especially when you are exposed to stressful conditions or painful experiences.	9.11
Practice of the tree.	This practice of guided imagery is inspired by the stability, strength, and openness of the tree, which is rooted in the earth. This practice favors the engagement with the present moment and therefore helps to overcome the rumination that is oriented towards the past or the future.	7.22
Practice of kindness towards yourself.	This practice helps to manage the emotional discomfort resulting from specific painful events or stressors. This practice favors balance and acceptance of one’s own experience.	13.16
Mindfulness practice for healthcare professionals.	This practice is useful to relieve tension and physical fatigue of those who work in the healthcare field. By focusing on the breath, this practice favors self-care and the connection with the present moment and with oneself.	13.32

**Table 2:** Sociodemographic characteristics and COVID-19 experience of the responding employees.

Variables	N (%)
Sex	
Male	62 (20)
Female	246 (80)
Age (years)	
Mean (SD)	45.06 (11.34)
Median (range)	47 (22–67)
Profession	
Administrative	48 (16)
Physician	48 (16)
Nurses	111 (36)
Allied healthcare professional	18 (6)
Psychologist	43 (14)
Physiotherapist	10 (3)
Health and safety officer	5 (2)
Biologist/Lab Technician	12 (3)
Other	13 (4)
Contact with COVID-19 patients	
Yes	160 (52)
No	148 (48)
Had COVID-19	
Yes	28 (9)
No	280 (91)
Family members with COVID-19	
Yes	37 (12)
No	271 (88)
Losses for COVID-19 in the personal context	
Yes	47 (15)
No	261 (85)
Losses for COVID-19 at work	
Yes	124 (40)
No	184 (60)

**Table 3:** Association of sex, age, profession and COVID-19 experience with anxiety (STAI-Y1), depression (HAM-D) and post-traumatic stress disorder (PCL-5).

	STAI-Y1				HAM-D				PCL-5			
	Unadjusted	Adjusted			Unadjusted		Adjusted		Unadjusted		Adjusted	
Variable	OR	(95% C.I.)	OR	(95% C.I.)	OR	(95% C.I.)	OR	(95% C.I.)	OR	(95% C.I.)	OR	(95% C.I.)
Sex (F vs. M)	1.05	(0.54–2.03)	1.14	(0.56–2.23)	2.75 **	(1.44–5.25)	2.82 *	(1.43–5.59)	1.17	(0.62–2.21)	1.17	(0.59–2.34)
Age (Years)	1.02	(0.99–1.04)	1.01	(0.98–1.04)	1.01	(0.99–1.03)	1.02	(0.99–1.04)	1.01	(0.99–1.04)	1.01	(0.99–1.04)
Profession												
Administrative ^1^	1	-	1	-	1	-	1	-	1	-	1	-
Physician	0.92	(0.43–2.00)	0.82	(0.35–1.91)	0.96	(0.48–1.91)	0.94	(0.43–2.03)	0.66	(0.31–1.39)	0.87	(0.37–2.02)
Other healthcare prof	1.45	(0.71–2.95)	1.41	(0.65–3.08)	0.75	(0.37–1.52)	0.62	(0.28–1.36)	0.83	(0.40–1.76)	0.58	(0.25–1.32)
Contact with COVID-19 patients (Yes vs. No)	1.08	(0.64–1.85)	0.99	(0.51–1.93)	0.95	(0.58–1.56)	1.03	(0.51–1.96)	1.71 *	(1.01–2.90)	1.44	(0.72–2.86)
Had COVID-19 (Yes vs. No)	0.70	(0.25–1.91)	0.59	(1.95–1.79)	0.88	(0.37–2,11)	0.80	(0.29–2.24)	2.73 *	(1.08–6.86)	1.63	(0.56–4.76)
Family member with COVID-19 (Yes vs. No)	1.54	(0.72–3.31)	1.24	(0.50–3.05)	1.47	(0.68–3.18)	1.46	(0.56–3.80)	2.86 *	(1.28–6.38)	2.75 *	(1.01–7.48)
Losses for COVID-19 in the personal context (Yes vs. No)	2.70 **	(1.40–5.21)	2.40 *	(1.16–4.98)	1.18	(0.59–2.34)	1.04	(0.47–2.31)	0.89	(0.42–1.86)	0.59	(0.24–1.44)
Losses for COVID-19 at work (Yes vs. No)	1.10	(0.64–1.88)	1.04	(0.57–1.91)	1.38	(0.84–2.29)	1.60	(0.90–2.85)	1.96 *	(1.15–3.33)	1.76	(0.97–3.19)

^1^ Reference category; * *p* < 0.05; ** *p* < 0.01.

## Data Availability

The data presented in this study are available on request from the corresponding author. The data are not publicly available due to ethical restrictions.

## References

[1] Sorbello M., El-Boghdadly K., Di Giacinto I., Cataldo R., Esposito C., Falcetta S., Merli G., Cortese G., Corso R.M., Bressan F. (2020). The Italian coronavirus disease 2019 outbreak: Recommendations from clinical practice. Anaesthesia.

[2] Pappa S., Ntella V., Giannakas T., Giannakoulis V.G., Papoutsi E., Katsaounou P. (2020). Prevalence of depression, anxiety, and insomnia among healthcare workers during the COVID-19 pandemic: A systematic review and meta-analysis. Brain. Behav. Immun..

[3] Vizheh M., Qorbani M., Arzaghi S.M., Muhidin S., Javanmard Z., Esmaeili M. (2020). The mental health of healthcare workers in the COVID-19 pandemic: A systematic review. J. Diabetes Metab. Disord..

[4] Spoorthy M.S., Pratapa S.K., Mahant S. (2020). Mental health problems faced by healthcare workers due to the COVID-19 pandemic–A review. Asian J. Psychiatr..

[5] Lai J., Ma S., Wang Y., Cai Z., Hu J., Wei N., Wu J., Du H., Chen T., Li R. (2020). Factors associated with mental health outcomes among health care workers exposed to Coronavirus Disease 2019. JAMA Netw. Open.

[6] Lu W., Wang H., Lin Y., Li L. (2020). Psychological status of medical workforce during the COVID-19 pandemic: A cross-sectional study. Psychiatry Res..

[7] Zhou Y., Wang W., Sun Y., Qian W., Liu Z., Wang R., Qi L., Yang J., Song X., Zhou X. (2020). The prevalence and risk factors of psychological disturbances of frontline medical staff in china under the COVID-19 epidemic: Workload should be concerned. J. Affect. Disord..

[8] Liu C.-Y., Yang Y., Zhang X.-M., Xu X., Dou Q.-L., Zhang W.-W. (2020). The prevalence and influencing factors for anxiety in medical workers fighting COVID-19 in China: A cross-sectional survey. SSRN Electron. J..

[9] Di Tella M., Romeo A., Benfante A., Castelli L. (2020). Mental health of healthcare workers during the COVID-19 pandemic in Italy. J. Eval. Clin. Pract..

[10] Rossi R., Socci V., Pacitti F., Di Lorenzo G., Di Marco A., Siracusano A., Rossi A. (2020). Mental health outcomes among frontline and second-line health care workers during the Coronavirus disease 2019 (COVID-19) pandemic in Italy. JAMA Netw. Open.

[11] Liang Y., Chen M., Zheng X., Liu J. (2020). Screening for Chinese medical staff mental health by SDS and SAS during the outbreak of COVID-19. J. Psychosom. Res..

[12] Xu J., Xu Q., Wang C., Wang J. (2020). Psychological status of surgical staff during the COVID-19 outbreak. Psychiatry Res..

[13] Lasalvia A., Bonetto C., Porru S., Carta A., Tardivo S., Bovo C., Ruggeri M., Amaddeo F. (2020). The psychological impact of the COVID-19 pandemic on health care workers in a highly burdened area of north-east Italy. Epidemiol. Psychiatr. Sci..

[14] Dubey S., Biswas P., Ghosh R., Chatterjee S., Dubey M.J., Chatterjee S., Lahiri D., Lavie C.J. (2020). Psychosocial impact of COVID-19. Diabetes Metab. Syndr. Clin. Res. Rev..

[15] De Kock J.H., Latham H.A., Leslie S.J., Grindle M., Munoz S.A., Ellis L., Polson R., O’Malley C.M. (2021). A rapid review of the impact of COVID-19 on the mental health of healthcare workers: Implications for supporting psychological well-being. BMC Public Health.

[16] Santabárbara J., Bueno-Notivol J., Lipnicki D.M., Olaya B., Pérez-Moreno M., Gracia-García P., Idoiaga-Mondragon N., Ozamiz-Etxebarria N. (2021). Prevalence of anxiety in health care professionals during the COVID-19 pandemic: A rapid systematic review (on published articles in Medline) with meta-analysis. Prog. Neuro-Psychopharmacol. Biol. Psychiatry.

[17] Tan B.Y.Q., Chew N.W.S., Lee G.K.H., Jing M., Goh Y., Yeo L.L.L., Zhang K., Chin H.-K., Ahmad A., Khan F.A. (2020). Psychological impact of the COVID-19 pandemic on health care workers in Singapore. Ann. Intern. Med..

[18] Li Z., Ge J., Yang M., Feng J., Qiao M., Jiang R., Bi J., Zhan G., Xu X., Wang L. (2020). Vicarious traumatization in the general public, members, and non-members of medical teams aiding in COVID-19 control. Brain. Behav. Immun..

[19] Eftekhar Ardebili M., Naserbakht M., Bernstein C., Alazmani-Noodeh F., Hakimi H., Ranjbar H. (2020). Healthcare providers experience of working during the COVID-19 pandemic: A qualitative study. Am. J. Infect. Control.

[20] Szmyd B., Karuga F.F., Bartoszek A., Staniecka K., Siwecka N., Bartoszek A., Błaszczyk M., Radek M. (2021). Attitude and behaviors towards SARS-CoV-2 vaccination among healthcare workers: A cross-sectional study from Poland. Vaccines.

[21] Smereka J., Szarpak L. (2020). The use of personal protective equipment in the COVID-19 pandemic era. Am. J. Emerg. Med..

[22] DiBenigno J., Kerrissey M. (2020). Structuring mental health support for frontline caregivers during COVID-19: Lessons from organisational scholarship on unit-aligned support. BMJ Lead..

[23] Weibelzahl S., Reiter J., Duden G. (2021). Depression and anxiety in healthcare professionals during the COVID-19 Pandemic. Epidemiol. Infect..

[24] Luo M., Guo L., Yu M., Jiang W., Wang H. (2020). The psychological and mental impact of coronavirus disease 2019 (COVID-19) on medical staff and general public—A systematic review and meta-analysis. Psychiatry Res..

[25] Rosenstein A.H. (2019). Hospital administration response to physician stress and burnout. Hosp. Pract..

[26] Muller A.E., Hafstad E.V., Himmels J.P.W., Smedslund G., Flottorp S., Stensland S.Ø., Stroobants S., Van de Velde S., Vist G.E. (2020). The mental health impact of the covid-19 pandemic on healthcare workers, and interventions to help them: A rapid systematic review. Psychiatry Res..

[27] Spielberger C.D., Gorsuch R.L., Lushene R., Vagg P.R., Jacobs G.A. (1983). Manual for the State-Trait Anxiety Inventory STAI (Form Y).

[28] Pedrabissi L., Santiniello M. (1989). Manuale Dell’adattamento Italiano Dello STAI Forma Y..

[29] Barisone M.G., Lerda S., Ansaldi S., De Vincenzo E., Angelini G. (2004). Psychopathology and epilepsy: Clinical experience in a Centre for the Diagnosis and Care of epilepsy. J. Psychopatol..

[30] Hamilton M. (1960). A Rating Scale for Depression. J. Neurol. Neurosurg. Psychiatry.

[31] Pancheri P., Picardi A., Pasquini M., Gaetano P., Biondi M. (2002). Psychopathological dimensions of depression: A factor study of the 17-item Hamilton depression rating scale in unipolar depressed outpatients. J. Affect. Disord..

[32] Zimmerman M., Martinez J.H., Young D., Chelminski I., Dalrymple K. (2013). Severity classification on the Hamilton depression rating scale. J. Affect. Disord..

[33] Blevins C.A., Weathers F.W., Davis M.T., Witte T.K., Domino J.L. (2015). The Posttraumatic Stress Disorder Checklist for DSM-5 (PCL-5): Development and initial psychometric evaluation. J. Trauma. Stress.

[34] American Psychiatric Association (2013). Diagnostic and Statistical Manual of Mental Disorders (DSM 5).

[35] Weathers F.W., Litz B.T., Keane T.M., Palmieri P.A., Marx B.P., Schnurr P.P. (2013). The PTSD Checklist for DSM-5 (PCL-5). https://www.ptsd.va.gov/professional/assessment/adult-sr/ptsd-checklist.asp#obtain.

[36] Bovin M.J., Marx B.P., Weathers F.W., Gallagher M.W., Rodriguez P., Schnurr P.P., Keane T.M. (2016). Psychometric properties of the PTSD Checklist for Diagnostic and Statistical Manual of Mental Disorders–Fifth Edition (PCL-5) in veterans. Psychol. Assess..

[37] IBM Corp (2019). IBM SPSS Statistics for Windows, Version 26.0. 2019.

[38] Lissoni B., Del Negro S., Brioschi P., Casella G., Fontana I., Bruni C., Lamiani G. (2020). Promoting resilience in the acute phase of the COVID-19 pandemic: Psychological interventions for intensive care unit (ICU) clinicians and family members. Psychol. Trauma Theory Res. Pract. Policy.

[39] Buselli R., Baldanzi S., Corsi M., Chiumiento M., Del Lupo E., Carmassi C., Dell’Osso L., Cristaudo A. (2020). Psychological care of health workers during the COVID-19 Outbreak in Italy: Preliminary report of an Occupational Health Department (AOUP) responsible for monitoring hospital staff condition. Sustainability.

[40] Tomlin J., Dalgleish-Warburton B., Lamph G. (2020). Psychosocial support for healthcare workers during the COVID-19 pandemic. Front. Psychol..

[41] Giusti E.M., Pedroli E., D’Aniello G.E., Stramba Badiale C., Pietrabissa G., Manna C., Stramba Badiale M., Riva G., Castelnuovo G., Molinari E. (2020). The Psychological Impact of the COVID-19 Outbreak on Health Professionals: A Cross-Sectional Study. Front. Psychol..

[42] DeWolfe D.J., Nordboe D. (2000). Training Manual for Mental Health and Human Service Workers in Major Disasters.

[43] Green B.L., Wilson J.P., Lindy J.D., Figle C. (1985). Conceptualizing Post-traumatic stress disorder: A Psychosocial Framework. Trauma and its Wake, Volume I: The Study and Treatment of Post-Traumatic Stress Disorder.

[44] Myers D., Zunin H.S., Zunin L. (1990). Grief: The art of coping with tragedy. Today’s Superv..

[45] Menichetti Delor J.P., Borghi L., Cao di San Marco E., Fossati I., Vegni E. (2021). Phone follow up to families of COVID-19 patients who died at the hospital: Families’ grief reactions and clinical psychologists’ roles. Int. J. Psychol..

[46] Gesi C., Carmassi C., Cerveri G., Carpita B., Cremone I.M., Dell’Osso L. (2020). Complicated grief: What to expect after the Coronavirus pandemic. Front. Psychiatry.

[47] Eaton N.R., Keyes K.M., Krueger R.F., Balsis S., Skodol A.E., Markon K.E., Grant B.F., Hasin D.S. (2012). An invariant dimensional liability model of gender differences in mental disorder prevalence: Evidence from a national sample. J. Abnorm. Psychol..

[48] Nolen-Hoeksema S. (2012). Emotion regulation and psychopathology: The role of gender. Annu. Rev. Clin. Psychol..

[49] Szmyd B., Bartoszek A., Karuga F.F., Staniecka K., Błaszczyk M., Radek M. (2021). Medical Students and SARS-CoV-2 Vaccination: Attitude and Behaviors. Vaccines.

[50] Haller M., Norman S.B., Davis B.C., Capone C., Browne K., Allard C.B. (2020). A model for treating COVID-19-related guilt, shame, and moral injury. Psychol. Trauma Theory Res. Pract. Policy.

[51] Shanafelt T., Ripp J., Trockel M. (2020). Understanding and addressing sources of anxiety among health care professionals during the COVID-19 pandemic. JAMA.

[52] Cox C.L. (2020). ‘Healthcare Heroes’: Problems with media focus on heroism from healthcare workers during the COVID-19 pandemic. J. Med. Ethics.

